# Cefradine blocks solar-ultraviolet induced skin inflammation through direct inhibition of T-LAK cell-originated protein kinase

**DOI:** 10.18632/oncotarget.8260

**Published:** 2016-03-22

**Authors:** Xiaoming Fan, Qiuhong Duan, Changshu Ke, Guiping Zhang, Juanjuan Xiao, Dan Wu, Xiaoyu Zeng, Jingwen Chen, Jinguang Guo, Jie Zhou, Fei Shi, Feng Zhu

**Affiliations:** ^1^ Department of Biochemistry and Molecular Biology, Tongji Medical College, Huazhong University of Science and Technology, Wuhan, 430030, PR China; ^2^ Department of Pathology, Tongji hospital, Huazhong University of Science and Technology, Wuhan, 430030, PR China; ^3^ Shanghai Biyi Chemical Science and Technology Co., Shanghai, 201203, PR China; ^4^ Department of Dermatology of the General Hospital of Air Force, Beijing, 100142, PR China

**Keywords:** cefradine, TOPK, signal transduction pathway, solar UV, skin inflammation

## Abstract

Skin inflammation, and skin cancer induced by excessive solar ultraviolet (SUV) is a great threat to human health. SUV induced skin inflammation through activating p38 mitogen-activated protein kinase (p38) and c-Jun N-termeinal kinases (JNKs). T-LAK cell-originated protein kinase (TOPK) plays an important role in this process. Herein, the clinical data showed TOPK, phospho-p38, phospho-JNKs were highly expressed in human solar dermatitis. *Ex vivo* studies showed that SUV induced the phosphorylation of p38 and JNKs in HaCat and JB6 cells in a dose and time dependent manner. Molecule docking model indicated cefradine, an FDA-approved cephalosporin antibiotic, directly binds with TOPK. The result of *in vitro* binding assay verified cefradine can directly bind with TOPK. *In vitro* kinase results showed cefradine can inhibit TOPK activity. *Ex vivo* studies further showed cefradine inhibited SUV-induced the phosphorylation level of p38, JNKs and H2AX through inhibiting TOPK activity in a dose and time dependent manner, and cefradine inhibited the secretion of IL6 and TNF-α in HaCat and JB6 cells. *In vivo* studies showed that cefradine down-regulated SUV-induced the phosphorylation of p38, JNKs and H2AX and inhibited the secretion of IL6 and TNF-α in Babl/c mice. These results indicated that cefradine can inhibit SUV-induced skin inflammation by blocking TOPK signaling pathway, and TOPK is an effective target for suppressing inflammation induced by SUV irradiation.

## INTRODUCTION

Solar ultraviolet (SUV) radiation includes UVA (315-400 nm), UVB (280-315 nm) and UVC (200-280 nm). All of UVC, 90% UVB and 10% UVA irradiation are absorbed by the ozone layer [1]. UVA exposure causes oxidative DNA modifications, and UVB results in the formation of cyclobutane pyrimidine dimers [2]. Thus, UVA and UVB are considered to be a major contributor to skin inflammation and cancer. SUV comprises approximately 95% UVA and 5% UVB. Although many pathways have been studied using pure UVA and UVB irradiation, the pathological change induced by SUV has not been well understood sufficient for the disease of prevention and treatment. SUV-induced oxidative stress is associated with some chronic inflammation diseases of skin, such as solar dermatitis, psoriasis and skin cancer [3]. Thus, suppression of the signaling pathway of inflammation was an effective strategy to manage SUV-induced skin diseases. p38 and JNKs pathway is a key regulator of proinflammatory cytokine biosynthesis at the transcriptional and translational levels [4–6]. And, p38α and JNKs can be activated strongly in SUV radiation induced inflammation [7].

T-LAK cell-originated protein kinase (TOPK), a newly identified member of the MEK3/6-related MAPKK family, is the up-stream kinase of p38 and JNKs [8–9]. It involves in mitotic checkpoint of tumor cell, DNA damage, tumor transformation and inflammation [10]. Previous studies suggested that TOPK contributed to p38 activation and JNKs phosphorylation during the UV-induced DNA damage response [9, 11]. TOPK can phosphorylate H2AX at Ser139 site, which had often been used as a biomarker of DNA damage [12–13]. Thus TOPK may be a potential target for chemotherapeutic or chemopreventive compounds in SUV-induced DNA damage and skin inflammation.

Currently, only two TOPK inhibitors, HI032 and OTS514 were discovered [14–15]. However, these compounds have great side effect and cannot be used clinically to treat disease. In this study, structure based virtual ligand screening method was employed to screen FDA-approved drug database. Cefradine, a FDA-approved first-generation broad-spectrum cephalosporin antibiotic is a TOPK inhibitor and suppresses the SUV-induced skin inflammation.

## RESULTS

### TOPK, phosphorylation of p38 and JNKs are overexpressed in human solar dermatitis

**Figure 1:**
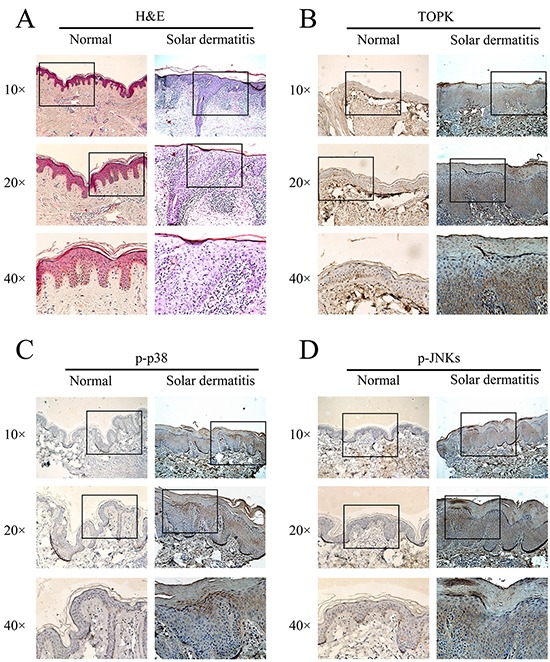
TOPK, phosphorylation of p38 and JNKs are over-expressed in human solar dermatitis **A.** The pathological change of solar dermatitis was detected by H&E. **B, C.** and **D.** Representative immunostaining results for TOPK, phospho-p38 and phospho-JNKs in human solar dermatitis (n=8) and normal skin tissues (n=6).

SUV inflicts inflammatory and oxidative damage on skin, which can lead to sunburn, photoaging, and photocarcinogenesis [17]. Previous studies of human and mouse models suggested that sunburn apoptosis, inflammatory cytokine induction and erythema were maximal following an acute early phase exposure to UV, and these acute responses are likely associated with unrepaired DNA damage [18]. SUV-induced inflammatory micro-environment appears to be a promoting event during solar dermatitis developing into skin cancers. Eight cases of solar dermatitis and six cases of normal skin samples were collected. The H&E results showed that the hyperkeratosis, thickness of epidermis, and infiltration of inflammatory cells in solar dermatitis were higher compared with the normal skin. The IHC results showed that the expression of phospho-JNKs (Figure 1B), phospho-p38 (Figure 1C) and TOPK (Figure 1D) in solar dermatitis were higher than that in the normal skin, and the data were summarized in Table 1. Next, we investigated the relationship between TOPK signaling pathway and SUV-induced dermatitis *in vivo* and *in vitro.*

**Table 1:** The positive staining of TOPK, p-p38, and p-JNKs in solar dermatitis cases (%)

Antigen		n	positive staining (%)
TOPK	solar dermatitis	8	7 (88)
	normal skin	6	1 (17)
p-p38	solar dermatitis	8	6 (75)
	normal skin	6	2 (33)
p-JNKs	solar dermatitis	8	5 (63)
	normal skin	6	1 (17)

### SUV induces the phosphorylation of the p38 and JNKs in a dose and time dependent manner in HaCat and JB6 cells

**Figure 2:**
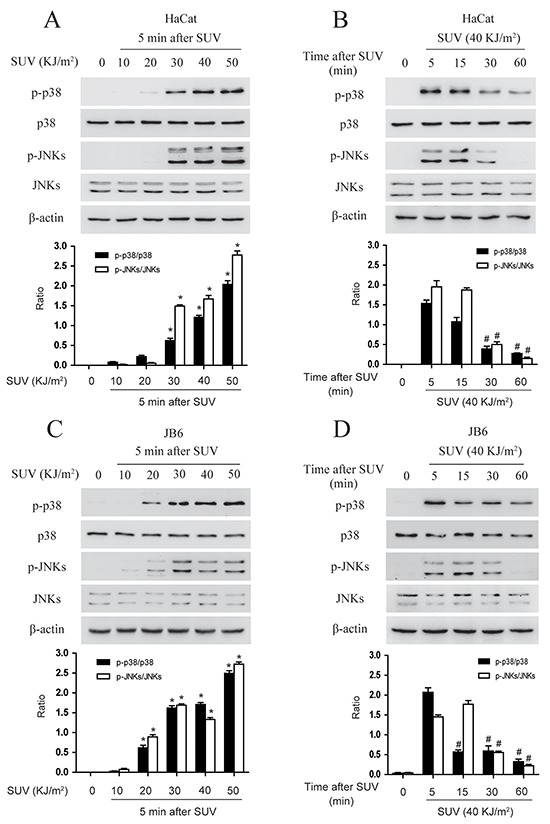
SUV induces the phosphorylation of the p38, JNKs in a dose and time dependent manner in the HaCat and JB6 cells **A.** HaCat cells (1×10^6^) were cultured in 10-cm dishes for 24 h, and then incubated with DMEM medium without serum for 12 h and treated with different dose of SUV as indicated. The cells then were replaced an incubator, and were harvested at 5 min after SUV. **B.** HaCat cells (1×10^6^) were cultured in 10-cm dishes for 24 h, and then incubated with DMEM medium without serum for 12 h and treated with 40KJ/m^2^ SUV, The cells were replaced in the incubator for different time point after 40KJ/m^2^ SUV, then were harvested. The cell lysates (30μg) were subjected to 10% SDS-PAGE. Protein bands were detected by Western blot. **C.** and **D.** JB6 cells were treated in the same manner with HaCat. Histogram shown are representative of least three independent experiments. The asterisks (*) indicate a significant (*P<0.05*) difference compared to 0 group. The pound (^#^) indicate a significant (*P<0.05*) difference compared to the group of 5 min after SUV exposure.

The activation of p38 and JNKs is an indicator of cellular stress after exposure of SUV radiation. Firstly, the phosphorylation level of p38 and JNKs were examined in HaCat and JB6 cells. To examine the optimal dose-time point at which SUV might stimulate a stress response in HaCat cells, the cells were exposed different dosage of SUV for the different time. The results indicated that phosphorylation level of p38 and JNKs were increased in a dose and time dependent manner. For example, the phosphorylation level of p38 and JNKs gradually increased from 10 to 50 KJ/m^2^ SUV (Figure 2A). Moreover, the phospho-p38 and phospho-JNKs level were up to highest at 5 min and cells maintained a healthy condition after high dose of SUV (40KJ/m^2^) exposure (Figure 2B). The similar results were observed in JB6 cells except that the phospho-JNKs level was highest at 15 min after exposure to 30 KJ/m^2^ SUV (Figure 2C and 2D). Because p38 activation was the dominant phenomena in SUV-induced inflammation signaling pathway [11], therefore, these two group cells were treated with 40 KJ/m^2^ SUV, the samples were collected 5 min later after SUV irradiation for the following experiment. The time point selection was based on Figure 2.

### TOPK knockdown inhibits SUV-induced the phosphorylation of p38 and JNKs in HaCat cells

**Figure 3:**
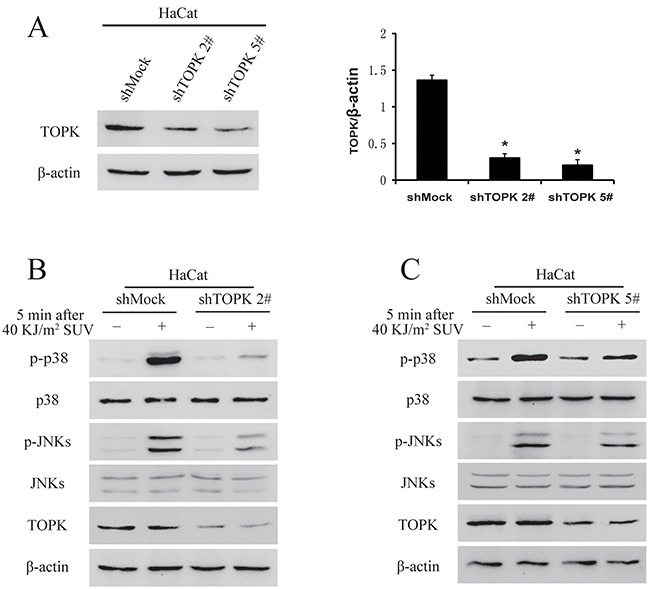
Knock down TOPK inhibits SUV-induced the phosphorylation of p38 and JNKs **A.** Efficiency of Knock down TOPK 2# and TOPK 5# in HaCat cells. Histogram shown are representative of least three independent experiments. The asterisks (*) indicate a significant (*P<0.05*) difference compared to shMock group. **B.** TOPK 2# inhibited the phosphorylation of p38 and JNKs significantly under the condition of SUV irradiation. HaCat shMock cells and HaCat shTOPK cells were stimulated with SUV light (40 KJ/m^2^) after 12 h of serum-free starvation. The whole cell lysates were extracted at 5 min after SUV light. The phosphorylation levels of p38, JNKs were detected by Western blot. **C.** Knock down TOPK 5# inhibited SUV-induced the phosphorylation of p38 and JNKs significantly.

Previous studies suggested that TOPK mediated UVB-induced p38α and JNKs activation [9, 19], and therefore TOPK knockdown may affect p38 activation after long-term growth-factor stimulation and reduces DNA damage response after UV irradiation in MCF7 cells [20]. TOPK was knocked down in HaCat cells, and stable cell lines infected with shTOPK-2 and shTOPK-5 were selected for further experiment (Figure 3A). The phosphorylation level of p38 and JNKs after 40 KJ/m^2^ SUV in shTOPK HaCat cells were dramatically decreased compared to shMock cells (Figure 3B). Therefore, the results showed that knockdown TOPK inhibited SUV induced the activity of p38 and JNKs.

### Cefradine directly binds with TOPK, and inhibits TOPK activity *in vitro* kinase assay

**Figure 4:**
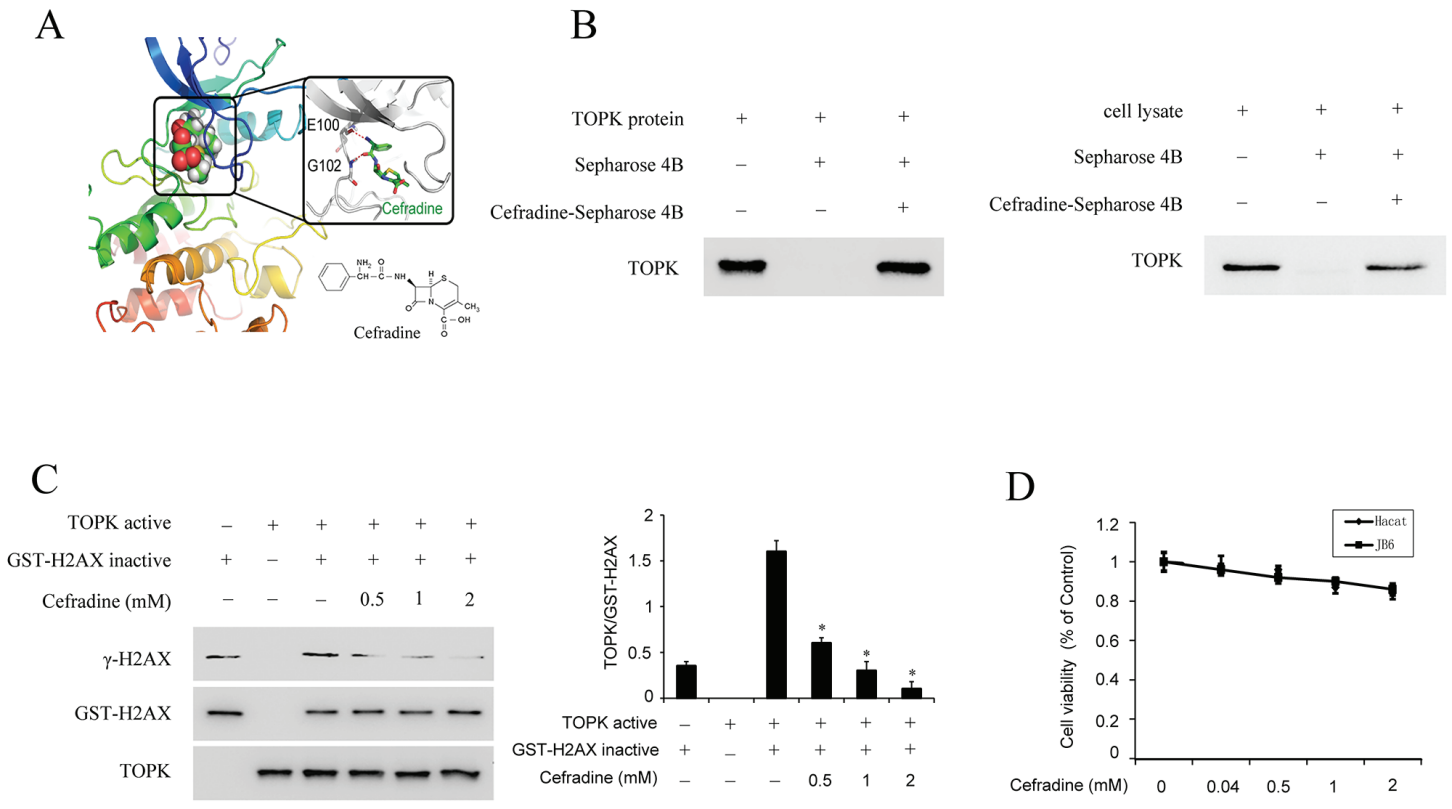
Cefradine binds with TOPK and inhibits TOPK activity **A.** The docking model of cefradine and chemical structure of cefradine. The docking model was described in ‘Materials and methods’. **B.** Cefradine binds directly with TOPK *ex vivo* and *in vitro*. Cefradine-Sepharose 4B or Sepharose 4B were used for binding and pull-down assay. **C.** Cefradine inhibits TOPK activity *in vitro* in a dose dependent manner. An inactive GST-H2AX protein was used as the substrate with active TOPK and 100 μM ATP. Protein were resolved by 10% SDS-PAGE gel and detected by Western blot. Histogram shown are representative of least three independent experiments. The asterisks (*) indicate a significant (*P<0.05*) difference compared to TOPK kinase and H2AX substrate group (lane 3). **D.** Cefradine have no effects on the cell viability of HaCat and JB6 cells. HaCat cells and JB6 cells were treated with 0.04, 0.5, 1 and 2 mM of Cefradine for 24 h, and measured using an MTS according to the manufacturer's instructions. Cells viability was measured and expressed as mean ± SEM at least three independent experiments.

The drug reposition of FDA-approved compounds has increased in recent years because of high cost of drug development. Next, structure based virtual screening was employed to identify TOPK inhibitor. Cefradine, an FDA-approved first-generation broad-spectrum cephalosporin antibiotic was identified as the top one of the TOPK inhibitor candidates. To estimate whether cefradine binds to TOPK, the homology modeling and subsequent molecular docking method were applied. The binding model generated by docking simulation indicated that the ATP binding pocket of TOPK was able to accommodate cefradine which forms two potential hydrogen bonds with the hinge residues E100 and G102. The hydrophobic moiety of cefradine inserted into the hydrophobic pocket while the hydrophilic end stayed at the solution-exposed pocket entrance (Figure 4A). To further evaluate this binding model, an *in vitro* binding assay was performed using cefradine-conjugated beads with purified TOPK protein and HaCat cell lysates. A strong band representing TOPK was observed in cefradine-conjugated beads group, whereas no obvious band was seen in beads alone group (Figure 4B). These results indicated that cefradine could bind directly to TOPK, suggesting that cefradine might inhibit the TOPK activity.

To confirm this hypothesis, an *in vitro* kinase assay was performed using GST-H2AX as substrate with active TOPK in the presence of 0.5, 1, 2 mM of cefradine. The result indicated that the phosphorylation level of HA2X was gradually decreased with increasing concentration of cefradine pre-treatment (Figure 4C).

Next, the cytotoxicity of cefradine was evaluated in HaCat and JB6 cells by MTS assay. Cefradine did not decrease the viability of the cells in the presence of cefradine (0.04, 0.5, 1, 2 mM) for 24 h (Figure 4D). Hence, cefradine does not have significant cytotoxicity on HaCat and JB6 cells.

### Cefradine down-regulates SUV-induced the downstream signaling pathway of TOPK by a dose and time dependent manner and cefradine inhibits SUV-induced the secretion of inflammatory factors in the HaCat and JB6 cells

**Figure 5:**
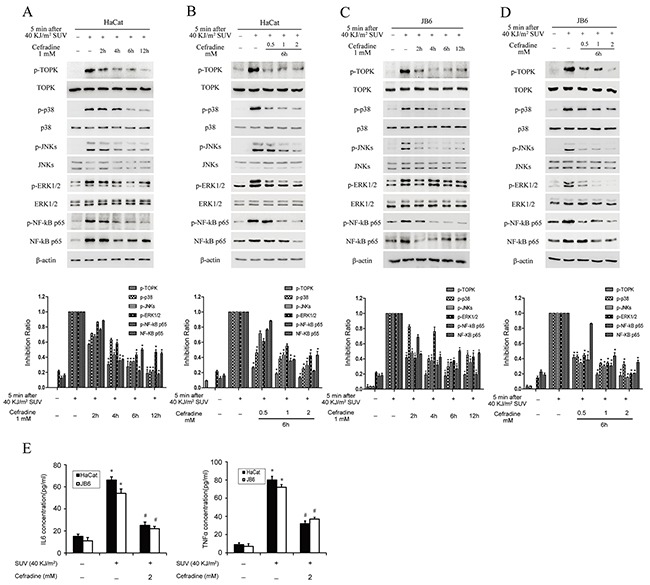
Cefradine down-regulates SUV-induced downstream TOPK signaling pathway in a dose and time dependent manner and inhibits the secretion of cytokines in the HaCat and JB6 cells **A.** HaCat cells (1×10^6^) were cultured in 10-cm dishes for 24 h, and then incubated with DMEM medium without serum for 12 h. Next, the cells were treated at 2, 4, 6, 12 h with 1 mM cefradine, then replaced in a incubator for 5 min after treated with 40KJ/m^2^ SUV. The cell lysates (30 μg) were subjected to 10% SDS-PAGE gel. Protein bands were detected by Western blot. **B.** HaCat cells (1×10^6^) were cultured in a 10-cm dishes for 24 h, and then incubated with DMEM medium without serum for 12 h and then treated for 6 h with 0.5, 1, 2 mM Cefradine. Then cells were replaced in incubator for 5 min after treated with 40KJ/m^2^ SUV. **C.** and **D.** JB6 were treated under the same conditions as for HaCat cells were. Histogram shown are representative of least three independent experiments. The asterisks (*) indicate a significant (*P<0.05*) difference compared to SUV alone group (lane 2). **E.** HaCat and JB6 cells were pre-treated for 6 h by 2 mM cefradine and stimulated with 40KJ/m^2^ SUV after 24 h of starvation, and the culture media was collected at 24 h. The concentrations of IL6 and TNF-α were determined using ELISA kits. The values shown are mean SEM of data from three independent experiments. The asterisks (*) indicate a significant (*P<0.05*) difference compared to control group. The pound (#) indicate a significant (*P<0.05*) difference compared to SUV group.

Next, we investigated whether cefradine could inhibit SUV-induced TOPK signaling pathway in HaCat and JB6 cells. Western blot results revealed that the level of phosphorylation of p38 and JNKs induced by SUV at 6 h or 12 h in HaCat cells were significantly decreased after pre-treated with different does of cefradine (Figure 5A and 5B). The phosphorylation level of p38 and JNKs in JB6 cells were further tested, and similar results were observed (Figure 5C and 5D). Previous studies showed that SUV can induce ERK1/2 and NF-κB activity [11, 21]. We detected the activity of ERK1/2 and NF-κB after SUV irradiation and cefradine pre-treatment, the results showed cefradine also suppressed SUV-induced the activity of ERK1/2 and NF-κB in HaCat and JB6 cells (Figure 5). Further, we detected that whether cefradine suppressed the secretion of IL6 and TNF-α. ELISA results showed that 2 mM cefradine significantly inhibited the secretion of IL6 and TNF-α in HaCat and JB6 cells (Figure 5E). These results suggested that cefradine inhibited the phosphorylation of downstream substrates of TOPK in a dose and time dependent manner and the secretion of inflammatory factors *ex vivo* under SUV treatment.

### Cefradine suppresses SUV-induced DNA damage in the HaCat and JB6 cells

**Figure 6:**
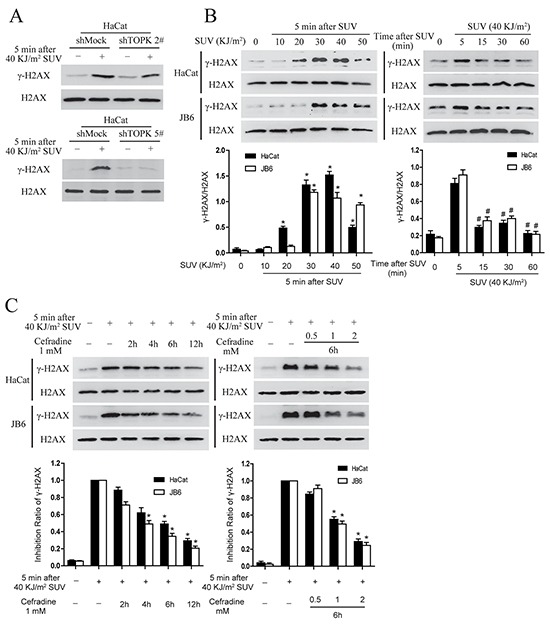
Cefradine suppresses SUV-induced DNA damage through inhibiting TOPK activity in a dose and time dependent manner in the HaCat and JB6 cells **A.** TOPK regulated the phosphorylation level of H2AX under the condition of 40KJ/m^2^ SUV in HaCat cells. HaCat shMock cells and HaCat shTOPK cells were stimulated with 40 KJ/m^2^ SUV after 12 h of serum-free starvation. The cells histones were extracted at 5 min after SUV irradiation. The phosphorylation levels of H2AX were detected by Western blot. **B.** SUV increased the phosphorylation of the H2AX in a dose and time dependent manner in the HaCat cells and JB6 cells. The cells were starvation for 12 h and then treated by different SUV dose and replaced in an incubator at different time points after 40 KJ/m^2^ SUV. The cells histones were extracted and the phosphorylation levels of H2AX were detected by Western blot. The asterisks (*) indicate a significant (*P<0.05*) difference compared to normal group. The pound (#) indicate a significant (*P<0.05*) difference compared to the group of 5 min after SUV exposure. **C.** Cefradine suppressed SUV-induced the phosphorylation of H2AX in a dose and time dependent manner in the HaCat and JB6 cells. The cells were starvation for 12 h and then treated at different time point using different concentration of cefradine under 40KJ/m^2^ SUV. The cells histones were extracted and subjected to 15% SDS-PAGE gel. Protein were detected by Western blot. Histogram shown are representative of least three independent experiments. The asterisks (*) indicate a significant (*P<0.05*) difference compared to SUV group.

H2AX is a substrate of TOPK [12]. In addition, knockdown TOPK also increased susceptibility to DNA damage and impaired production of γ-H2AX after SUV stimulation [20]. Therefore next, γ-H2AX was detected under SUV irradiation in shMock and shTOPK HaCat cell lines. The result showed that the level of γ-H2AX was decreased in shTOPK cells (Figure 6A). The level of γ-H2AX were tested in HaCat cells and JB6 cells after SUV irradiation, and the results showed that γ-H2AX was induced in a dose and time dependent manner (Figure 6B), suggesting DNA damage occurred under SUV treatment. To further verify whether cefradine could suppress SUV-induced DNA damage, different concentration of cefradine (0.5, 1, 2 mM) were used to treat the HaCat and JB6 cells at different time points (2, 4, 6, 12 h) under the condition of 40 KJ/m^2^ SUV stimulation. As demonstrated in Figure 6C, cefradine inhibited H2AX phosphorylation in a dose and time dependent manner in HaCat and JB6 cells.

### Cefradine inhibits inflammation induced by SUV irradiation in mouse skin

It has been reported that SUV irradiation induced skin inflammation with the increase of epidermis thickness and the secretion of inflammation factor [22–24]. To investigate the effect of cefradine on SUV-induced skin inflammation in mouse, adult Babl/c mice were sheared and smeared with cefradine (100 mg/kg), Three hours later, the hairless part of dorsal trunk skin was subjected to 100 KJ/m^2^ SUV irradiation. All the animals had been given euthanasia and the dorsal trunk skin were peeled away for H&E and IHC staining and ELISA assay 24 hours after SUV irradiation.

**Figure 7:**
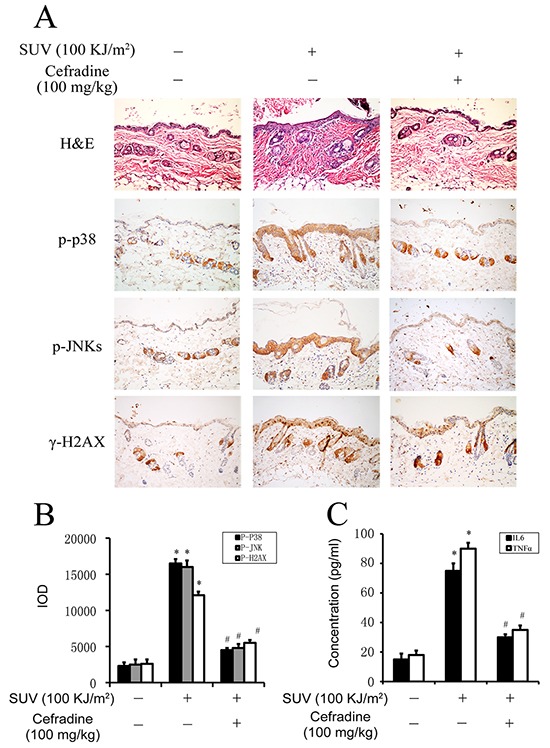
Cefradine inhibits inflammation induced by SUV irradiation in mouse skin **A.** Cefradine suppressed SUV-induced epidermis thicker and the proliferation of immunocytes. Adult Babl/c mice were irradiated with 100KJ/m^2^ SUV irradiation after cefradine smeared the dorsal skin for 3 h, and the skin were harvested at 24 h after irradiation and stained with H&E and IHC. **B.** Quantification of expression of phospho-p38, phospho-JNKs, and phospho-H2AX in mouse skin tissues were detected by the Image-Pro Plus software and data are shown as integrated optical density units. The values shown are the mean SEM of data from three independent experiments. **C.** Cefradine inhibited the secretion of IL6 and TNF-α induced by 100KJ/m^2^ SUV in mouse skin tissues. The concentration of IL6 and TNF-α were determined using ELISA kits. Significant differences were determined by one-way analysis of variance. The asterisks (*) indicate a significant (*P<0.01*) difference compared to control group. The pound (#) indicate a significant (*P<0.05*) difference compared to SUV group.

Our data revealed that the epidermis thickness increased significantly with the infiltration of immunocytes in the 100 KJ/m^2^ SUV compared with the normal control group (Figure 7A upper panel: middle column versus left column). The thickness of the epidermis of mice treated with 100 mg/kg cefradine were remarkably reduced, and the infiltration of immunocytes were also reduced compared with the SUV group by H&E detection (Figure 7A upper panel: right column versus middle column). Next, the TOPK downstream substrates were tested on paraffin section of skin samples. The phosphorylation level of p38, JNKs and H2AX were significantly increased after SUV irradiation, and the level were significantly inhibited after cefradine treatment by IHC staining respectively (Figure 7A). Quantification of expression of phospho-p38, phospho-JNKs, and γ-H2AX in mouse skin tissues in Figure 7A were analyzed by the Image-Pro Plus software, and the expression of phosphorylation of p38, JNKs and H2AX treated with 100 mg/kg cefradine were significantly suppressed by 82%, 89% and 72%, respectively, compared to the SUV group (Figure 7B, *P<0.01*). Furthermore, the secretion of the inflammatory factor IL6 and TNF-α induced by SUV in the mouse skin tissues were tested to check the effect of cefradine. The results showed that cefradine suppressed SUV-induced the secretion of IL6 and TNF-α in vivo (Figure 7C). In conclusion, these data indicated that cefradine could protect mouse skin from SUV irradiation induced inflammation response by inhibiting TOPK signaling pathway *in vivo*.

## DISCUSSION

SUV has been recognized as an environmental factor in the development of epidermis inflammation among men and women worldwide. The most common symptoms are skin inflamed and burn. The acute inflammation, in some cases, promotes chronic inflammation in a slow process, and at last evolves into skin carcinoma in several years [25]. Most skin diseases are related to UV irradiation, such as solar dermatitis, ichthyosis, psoriasis, actinic keratosis, and skin cancers.

TOPK has been known to be a serine/threonine mitogen-activated protein kinases-like (MAPK-like) protein kinase [26]. Previous studies indicated that TOPK was highly expressed in malignant melanoma tissues [27] and RPMI7951 melanoma cells [12]. TOPK was highly expressed in clinical samples of solar dermatitis (Figure 1D). It is well known that MAPK, such as p38 and JNKs are significant targets of SUV-induced inflammation and oxidative stress in skin [28–30]. SUV can induce the activity of ERK1/2 [11] and NF-kB [21]; TOPK can phosphorylate ERK1/2 [31] and activate NF-kB [32]. TOPK is an upstream kinase of p38 and JNKs. In this study, we found the phosphorylation level of p38 and JNKs also were highly expressed in solar dermatitis (Figure 1C and 1D). Therefore, TOPK is an ideal target for therapy for the skin inflammatory diseases caused by SUV irradiation.

Several chemotherapy agents for the above diseases have been shown to have some side effects, such as weakening the immune system [33–34]. It is necessary to find a drug of lower toxicity and specific targets to cure skin disease. Our study suggested a novel therapeutic strategy that different from traditional therapy for skin diseases, such as topical steroids. A stinging or burning feeling when you first apply the topical steroids, and the symptoms often reverses when the topical steroid is stopped. Side-effects from topical steroids may develop permanent stretch marks, bruising, discolouration, or thin spidery blood vessels. It may also trigger or worsen other skin disorders such as acne, rosacea and perioral dermatitis [35]. Because the topical steroids have no special target, it gets through the skin and into the bloodstream to cause systemic symptoms, such as fluid collection in the legs, high blood pressure, bone damage and Cushing's syndrome [36]. It is necessary to find non-steroid drugs targeting key players in the UV signaling pathway for skin diseases. Cefradine, an FDA-approved cephalosporin antibiotic, was used for treating inflammatory diseases caused by bacteria, but here, it can be a new molecule-targeted agent of low toxicity to cure skin diseases. The incidence of adverse reaction of cefradine is lower, and the adverse reaction is lighter than the topical steroids which have no special target. There were no significant toxicity to human health after cefradine was used so long time [37]. Our data clearly indicated that it manifested the faculty to attenuate inflammatory responses induced by SUV by targeting directly TOPK.

More than $800 million was spent in 10 to 17 years required for developing a drug *de novo* [38]. Still, there are also side effects or off targets to induce the drug development termination after entered the clinic [39]. The reposition of the drug approved by FDA has increased in recent years, because these types of drugs are more likely to enter clinical trials more quickly and cheaply. In this study, we identify cefradine as TOPK inhibitor by virtual screen method, and it may be a shortcut to identify new therapeutic agents for skin diseases caused by SUV irradiation. Cefradine can be a promising ingredient added in sun cream for preventing skin burn induced by SUV, and it can be used for curing SUV-induced skin diseases, such as solar dermatitis, psoriasis, actinic keratosis and skin cancer clinically.

## MATERIALS AND METHODS

### Solar-UV, reagents and antibodies

For this study, SUV lamps (UVA-340nm) were purchased from Q-Lab Corporation (Cleveland, OH). The percentage of UVA and UVB of SUV lamps was measured by a UV meter and was 92.5% and 7.5% respectively. Commercial cefradine was obtained from Sigma-Aldrich (St Louis, MO, USA). The active TOPK human protein for the kinase assay was purchased from Millippore (Billerica, MA, USA). The pGEX-GST-H2AX human plasmid were purchased from Addgene Inc. The IL6 and TNF-α ELISA kit were purchased from Biosen Inc. (Beijing, China). Antibodies to detect total TOPK, total JNKs, total p38, total ERK1/2, total NF-κB p65, total H2AX, phosphorylated JNKs (Thr183/Thr185), phosphorylated p38 (Thr180/Thr182), phosphorylated ERK1/2 (Thr202/Tyr204), phosphorylated NF-κB p65 (S536) and phosphorylated H2AX (Ser139) were purchased from Cell Signaling Technology (Billerica, MA, USA). Antibodies to detect β-actin were purchased from Santa Cruz Biotechnology (Santa Cruz, CA, USA).

### Cell culture and MTS assay

The human skin keratinocytes HaCat cell line and mouse epidermal JB6 Cl41 (JB6) cell line were purchased from ATCC, Virginia, USA. HaCat cells were cultured at 37°C in a 5% CO_2_ incubator in Dulbecco's modified Eagle's medium (DMEM) containing 10% fetal bovine serum (FBS). JB6 cells were cultured at 37°C in a 5% CO_2_ incubator in Eagle's minimum essential medium (MEM) supplemented with 5% FBS. Two kinds of cell lines were plated at 1×10^5^ cells per 6-cm dish and counted using a blood counting chamber.

Cells were seeded (8×10^3^ cells per well) in 96-well plates and cultured overnight, and then fed with fresh medium and treated with different concentration of cefradine. The cytotoxicity of cefradine was measured at different time points using an MTS (3-(4,5-dimethylthiazol-2-yl)-5-(3-carboxymethoxyphenyl)-2H-tetrazdium) assay kit (Promega, Madison, WI). The absorbance was read at 490 nm according to the instructions.

### Western blot

The cells (1×10^6^) were cultured in 10-cm dishes to 50% - 60% confluence. The cells were treated with various doses of SUV (0, 10, 20, 30, 40, 50 KJ/m^2^) and harvested at different times (0, 15, 30, 60 min). For studying the effect of cefradine, the cells remained starved in serum-free medium for 12 h, then treated with cefradine (0.5, 1, 2 mM) for 2, 4, 6, 12 h before treatment with SUV light. The harvested cells were disrupted and the protein concentrations of cells were determined by the Bradford method. The samples (20-30 μg) with 5×sodium dodecyl sulfate (SDS) loading buffer were heated at 95°C for 10 minutes. Next, the samples were loaded to 10% SDS polyacrylamide gel electrophoresis (SDS-PAGE) and transferred onto polyvinylidene fluoride membrane (PVDF) (Millipore, Billerica, MA). PVDF membrane was incubated with a specific primary antibody at 4°C overnight. Proteins were visualized using a chemiluminescence detection kit (BIO-RAD, USA) after hybridization with a horseradish peroxidase-conjugated secondary antibody.

### Bacterial expression and purification of the GST-H2AX fusion protein

The human GST-H2AX fusion protein was expressed in *E. Coli* BL21 bacteria. The bacteria were grown at 37°C to an absorbance of 0.8–0.9 at 600 nm, induced with 0.5 mM isopropyl-β-D-thiogalactopyranoside (IPTG) 2-3 h at 37°C, and then harvested by centrifugation. The cell pellets were suspended in Phosphate Buffered Saline (PBS). After sonication and centrifugation, the supernatant fraction was incubated with Glutathione-Sepharose beads (GE, USA) overnight at 4°C. The beads were washed with PBS and then eluted with 50 mM Glutathione. After protein quantitation, the samples were separated by 10% SDS-PAGE, and visualized by Coomassie Brilliant Blue staining.

### *In vitro* kinase assay

GST-H2AX (1 μg) proteins were used as the substrate for an *in vitro* kinase assay with 250 ng of active TOPK protein. Reactions were carried out in 1×kinase buffer (25 mM Tris-HCl pH 7.5, 5 mM β-glycerophosphate, 2 mM dithiothreitol (DTT), 0.1 mM Na_3_VO_4_, and 5 mM MnCl_2_) containing 100 μM ATP at 30°C for 30 min and they were stopped by 5×SDS loading buffer. Phosphorylated H2AX, total H2AX and total TOPK were detected by Western blot.

### Molecular modeling and docking simulation

To estimate the interaction mode of TOPK and cefradine, a TOPK structure was modeled and subsequent induced-fit docking was applied. The sequence of TOPK (GI:83305809) was downloaded from the NCBI, and Protein Basic Local Alignment Search Tool (BLAST) against the Protein Data Bank (PDB) was performed to identify homologous sequence. Among those with the highest sequence identity (30%) with TOPK, structures of 4L52, 2EVA, 4GS6 (PDB entries) were protein-ligand complex, thus suitable for the modeling of the TOPK-cefradine complex. An extra structure of PDB 2F4J with sequence identity of 27% with respect to TOPK was selected for the template group based on a previous study [14]. The sequence of TOPK and the four templates, 4L52, 2EVA, 4GS6 and 2F4J, were aligned using ClustalW2 server with the default parameters. The secondary structures of TOPK and the templates were predicted and compared by SSpro 4.0 server. The multiple sequence alignment was then used to build the homology model by MODELER 9v8 [16]. Ten models were generated and the structure with the best discrete optimized protein energy (DOPE) score was selected as the TOPK modeled structure. The reliability of the TOPK modeled structure was confirmed by SAVES server. The TOPK modeled structure was then submitted to the “protein preparation wizard” module within Maestro version 9.0 (Schrödinger, LLC, 2010, New York, NY) for optimization. The structure of cefradine was sketched and prepared using LigPre module of Maestro with the pH value in the range of 7.0±0.2. To generate the binding mode, the induced-fit docking method in Maestro version 9.0 was applied with residues lining the ATP binding pocket of TOPK selected as flexible residues.

### *In vitro* binding assay

HaCat cell lysates (1 mg) were incubated with cefradine-Sepharose 4B or Sepharose 4B alone in the reaction buffer [50 mM Tris (pH 7.5), 5 mM ethylenediaminetetraacetic acid (EDTA), 150 mM NaCl, 1 mM DTT, 0.01% Nonidet P-40, 2 μg/ml bovine serum albumin, 0.02 mM phenylmethylsulfonyl fluoride (PMSF) and 1 μg/ml protease inhibitor mixture]. After gentle rocking overnight at 4°C, the beads were washed five times, and then the beads were loaded to 10% SDS-PAGE with 2×SDS loading buffer and analyzed by Western blot.

### Animal study

Adult Babl/c mice at 6-8 weeks old were purchased from the Center for Disease Control (Hubei, China). The animals were allowed to rest for 5 days under the condition of normal natural conditions. The animal's dorsal hair were shaved 24 hours before experiment, and the hairless dorsal skin was irradiated with 100 KJ/m^2^ SUV light in the resting phase 3 hours after the dorsal skin being smeared with cefradine. The mice were euthanized and their dorsal trunk skin samples were harvested at 24 h after SUV irradiation. One-half of the samples were immediately fixed in 4% paraformaldehydeand for hematoxylin and eosin (H&E) staining and immunohistochemistry (IHC). The other samples were frozen and used for ELISA analysis. All animal experiments were carried out abiding by the rules of the Laboratory Animal Center of Tongji Medical College Huazhong University of Science and Technology.

### Immunohistochemistry

The tissue sections (5 μm) were performed antigen retrieval by microwave after deparaffinization and rehydration for 10 min in sodium citrate buffer. The sections were cooled to room temperature, treated with 3% H_2_O_2_ for 10 min and blocked with 5% goat serum for 40 min at room temperature. Next, the sections were incubated at 4°C for overnight with a primary antibodies. Then the sections were washed in PBS and incubated with the secondary antibody for 30 min. After PBS washing, the sections were incubated with 3,3-diaminobenzidine (DAB) as substrate for 3 minutes. For evaluation, photomicrographs were taken with a digital camera. The positively stained cells within each photomicrograph were counted.

### Statistical analysis

All quantitative data are expressed as mean values ± standard deviation, and significant differences were determined by Student's Test or by one-way ANOVA. A probability value of *P<0.05* was used as the criterion for statistical significance.
